# Identifying Gaps in the International Consensus Case Definitions for Invasive Aspergillosis: A Review of Clinical Cases Not Meeting These Definitions

**DOI:** 10.1093/ofid/ofae594

**Published:** 2024-10-09

**Authors:** Shio Yen Tio, Sharon C A Chen, Christopher H Heath, Alyssa Pradhan, Arthur J Morris, Tony M Korman, C Orla Morrissey, Catriona L Halliday, Sarah Kidd, Timothy Spelman, Nadiya Brell, Brendan McMullan, Julia E Clark, Katerina Mitsakos, Robyn P Hardiman, Phoebe C M Williams, Anita J Campbell, Justin Beardsley, Sebastiaan Van Hal, Michelle K Yong, Leon J Worth, Monica A Slavin

**Affiliations:** Department of Infectious Diseases, Peter MacCallum Cancer Centre, Melbourne, Australia; National Centre for Infections in Cancer, Melbourne, Australia; Department of Infectious Diseases, Royal Melbourne Hospital, Melbourne, Australia; Sir Peter MacCallum Department of Oncology, University of Melbourne, Melbourne, Australia; Centre for Infectious Diseases and Microbiology Laboratory Services, Institute of Clinical Pathology and Medical Research, New South Wales Health Pathology, Westmead Hospital, Sydney, Australia; School of Medicine, University of Sydney, New South Wales, Australia; Department of Microbiology, PathWest Laboratory Medicine, Murdoch, Australia; Department of Infectious Diseases, Fiona Stanley Hospital, Murdoch, Australia; Department of Medicine, University of Western Australia, Crawley, Australia; School of Medicine, University of Sydney, New South Wales, Australia; Department of Infectious Diseases and Microbiology, Royal Prince Alfred Hospital, New South Wales, Australia; Auckland City Hospital, Auckland, New Zealand; Monash University and Monash Health, Clayton, Australia; Department of Infectious Diseases, Alfred Health and Monash University, Melbourne, Australia; Centre for Infectious Diseases and Microbiology Laboratory Services, Institute of Clinical Pathology and Medical Research, New South Wales Health Pathology, Westmead Hospital, Sydney, Australia; School of Medicine, University of Sydney, New South Wales, Australia; National Mycology Reference Centre, Microbiology and Infectious Diseases, South Australia Pathology, Adelaide, Australia; School of Biological Sciences, Faculty of Science Engineering & Technology, University of Adelaide, Adelaide, Australia; Department of Health Services Research, Peter MacCallum Cancer Centre, Melbourne, Australia; Department of Clinical Neuroscience, Karolinska Institute, Stockholm, Sweden; The Wollongong Hospital, New South Wales, Australia; Department of Infectious Diseases, Sydney Children's Hospital, Randwick, Australia; School of Clinical Medicine, Faculty of Medicine and Health, University of New South Wales (UNSW), New South Wales, Australia; Infection Management Service, Queensland Children's Hospital, Children's Health Queensland, Brisbane, Australia; School of Clinical Medicine, Childrens Health Queensland Clinical Unit, University of Queensland, Queensland, Australia; Department of Infectious Disease and Microbiology, Royal North Shore Hospital, Sydney, Australia; Department of Infectious Disease and Microbiology, Royal North Shore Hospital, Sydney, Australia; Department of Infectious Diseases, Sydney Children's Hospital, Randwick, Australia; School of Public Health, Faculty of Medicine, The University of Sydney, New South Wales, Australia; University of Sydney Infectious Diseases Institute, New South Wales, Australia; Department of Infectious Diseases, Perth Children's Hospital, Western Australia, Australia; Wesfarmers Centre for Vaccines and Infectious Diseases, Telethon Kids Institute, University of Western Australia, Western Australia, Australia; University of Sydney Infectious Diseases Institute, New South Wales, Australia; Westmead Hospital, Western Sydney Local Health District, NSW Health, New South Wales, Australia; Westmead Institute for Medical Research, New South Wales, Australia; School of Medicine, University of Sydney, New South Wales, Australia; Department of Infectious Diseases and Microbiology, Royal Prince Alfred Hospital, New South Wales, Australia; Department of Infectious Diseases, Peter MacCallum Cancer Centre, Melbourne, Australia; National Centre for Infections in Cancer, Melbourne, Australia; Department of Infectious Diseases, Royal Melbourne Hospital, Melbourne, Australia; Sir Peter MacCallum Department of Oncology, University of Melbourne, Melbourne, Australia; Department of Infectious Diseases, Peter MacCallum Cancer Centre, Melbourne, Australia; National Centre for Infections in Cancer, Melbourne, Australia; Sir Peter MacCallum Department of Oncology, University of Melbourne, Melbourne, Australia; Department of Infectious Diseases, Peter MacCallum Cancer Centre, Melbourne, Australia; National Centre for Infections in Cancer, Melbourne, Australia; Department of Infectious Diseases, Royal Melbourne Hospital, Melbourne, Australia; Sir Peter MacCallum Department of Oncology, University of Melbourne, Melbourne, Australia

**Keywords:** host factors, invasive aspergillosis, mortality, not meeting EORTC-MSGERC, single positive *Aspergillus* PCR

## Abstract

**Background:**

International consensus definitions for invasive aspergillosis (IA) in research are rigorous, yet clinically significant cases are often excluded from clinical studies for not meeting proven/probable IA case definitions. To better understand reasons for the failure to meet criteria for proven/probable infection, we herein review 47 such cases for their clinical and microbiological characteristics and outcomes.

**Methods:**

Data on 47 cases that did not meet consensus IA definitions but were deemed significant were derived from a retrospective, observational, multicenter survey of 382 presumed IA cases across Australasia, of which findings of 221 proven/probable infections were recently published. The clinical, microbiological, and radiologic characteristics of these cases were analyzed. Mortality outcomes were compared with those of 221 proven/probable cases.

**Results:**

Of 47 cases studied, 15 lacked classical host factors; 22 exhibited only a single positive *Aspergillus* polymerase chain reaction result; 7 lacked typical IA radiologic findings on chest computed tomography; and 3 had borderline galactomannan optical density indices (<1.0 but ≥0.5) in bronchoalveolar lavage fluid. The median age of patients was 61 years (IQR, 52–68); 34 were male (72%). Seven patients (15%) required intensive care admission. All patients had lung as the primary site of infection. Antifungal treatment was initiated in 42 patients (89%). All-cause 90-day mortality was 33%, similar to the 30% mortality in the comparative cohort (n = 221).

**Conclusions:**

Our findings highlight the limitations of current consensus definitions for IA. Notably, the mortality of patients not meeting these definitions was similar to that of patients with proven/probable IA. Further studies, especially of patients with a single positive *Aspergillus* polymerase chain reaction result and those without host factors, are needed to determine if future consensus definitions may benefit from modifications.

## Teaser

This study examined invasive aspergillosis cases that did not meet international consensus definitions yet were treated as significant to better understand the reasons for their exclusion from consensus case definitions and to raise awareness of their potential clinical significance.

International consensus criteria to define cases of invasive aspergillosis (IA) and other invasive fungal diseases, such as those of the European Organization for Research and Treatment of Cancer–Mycoses Study Group Education and Research Consortium (EORTC-MSGERC) [1], were established for clinical trial purposes to facilitate recruitment and comparability among patients across studies. However, in clinical practice, clinicians often encounter patients who do not fulfill these criteria yet are diagnosed with clinically significant IA, which warrants antifungal treatment. These cases are often excluded from clinical trials, creating a gap between consensus definitions and evidence-based management of IA.

**Figure 1.**
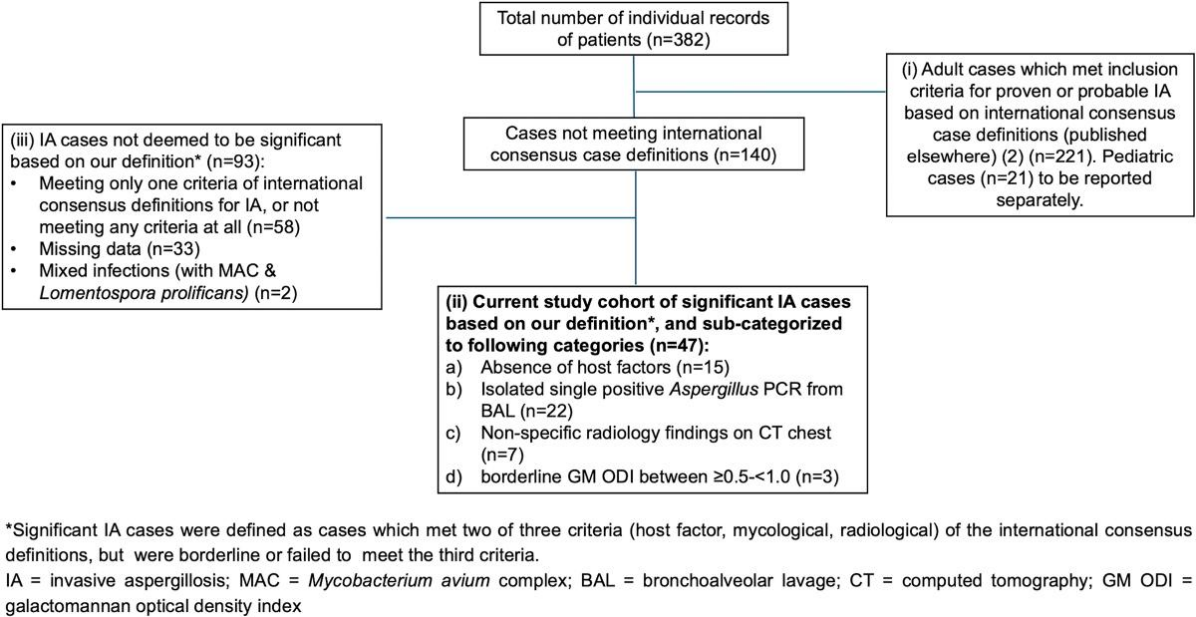
Flow diagram showing disposition of the invasive aspergillosis (IA) cases. *Significant IA cases were defined as cases that met 2 of 3 criteria (host factor, mycologic, radiologic) of the international consensus definitions but were borderline or failed to meet the third criterion. BAL, bronchoalveolar lavage; CT, computed tomography; GM ODI, galactomannan optical density index; MAC, *Mycobacterium avium* complex; PCR, polymerase chain reaction.

We recently conducted an in-depth clinical-epidemiologic analysis of 221 cases of proven and probable IA in adults across Australasia from January 2017 to December 2020 [2]. During the adjudication process for case inclusion, 47 of 382 (12%) captured cases were excluded for not meeting the internationally accepted definitions [1, 3–6] but nonetheless were significant and treated for IA (Figure 1). This prompted us to review these 47 cases, including their clinical, mycologic, and radiologic characteristics and outcomes, to better understand the reasons for their exclusion from consensus case definitions and to raise awareness of their potential clinical significance.

## METHODS

This retrospective, observational, multicenter cohort study involved 13 health care institutions across Australasia to evaluate the epidemiology and clinical outcomes of patients with proven and probable IA [2]. Each IA case in the database was reviewed by an adjudication committee and assigned as proven or probable IA based on recognized consensus definitions [1, 3–6]. Figure 1 shows the disposition of the cases and the justification for the numbers already reported (n = 221) [2] and for the present study (n = 47).

All 47 IA cases herein were considered significant, defined as having met 2 of 3 criteria (host factor, mycologic, radiologic) of the international consensus definitions but either were borderline or failed to meet the third criteria. These cases were deemed compelling as clinical IA by the treating clinician and adjudication committee, and the patients received directed antifungal treatment or palliation (if no treatment was offered). The cases were subcategorized into 4 groups according to reasons for not meeting consensus criteria:

Group a: absence of host factors
Group b: isolated/single positive *Aspergillus* polymerase chain reaction (PCR) result
Group c: lack of prespecified radiologic findings on chest computed tomography (CT)
Group d: borderline galactomannan (GM) optical density index (ODI) <1.0 but ≥0.5 in bronchoalveolar lavage fluid (BALF)

For all cases, clinical, mycologic, and radiologic characteristics were recorded, as well as antifungal treatment and outcomes (intensive care unit [ICU] admission and mortality). Mortality was evaluated at 30, 90, and 180 days from the time of diagnosis of significant IA cases or until death (whichever occurred sooner).

Descriptive analyses were performed with Stata version 16.1 (StataCorp LP) for patient demographics, the proportion of patients who received treatment, and mortality outcomes. All-cause mortality outcomes of these clinical IA cases were compared with the mortality outcomes in the previously studied cohort of 221 patients with proven/probable IA [2], in particular the 90-day mortality, which was the primary outcome for that study.

Human Research Ethics Committee approval (coordinating HREC 2019/ETH11822) was obtained at all study sites.

## RESULTS

Of a total of 382 IA cases captured in the database, 221 were previously reported [2]. Of the remaining, 47 were deemed to be significant IA cases based on our definition but did not fulfill the recognized consensus definitions for IA. These cases comprised patients as follows: group a, an absence of EORTC-MSGERC host factors (n = 15); group b, a single positive *Aspergillus* PCR result from BALF (n = 22); group c, nonspecific radiology findings on chest CT (n = 7); and group d, borderline GM ODI between ≥0.5 and <1.0 in BALF (n = 3). The remaining 93 cases were not significant according to our definition and were excluded for reasons outlined in Figure 1.

Details of the 47 cases are summarized in Supplementary Tables 1 to 4. The median age of patients was 61 years (IQR, 52–68). There were 34 males (72%) and 13 females (28%). Seven patients (15%) were admitted to ICU with a median duration of 8 days (IQR, 4–26). All 47 patients had the pulmonary site as the principal site of infection, with 1 patient having concurrent sinonasal involvement.

### Group a: IA Cases Without Host Risk Factors

Fifteen patients had mycologic and radiologic evidence suggestive of IA but did not have host factors as defined by EORTC-MSGERC definitions [1] (Supplementary Table 1).

Six patients had underlying solid organ malignancy, 5 of whom were not undergoing active therapy. One patient had active treatment (multikinase inhibitor lenvatinib), but this molecularly targeted therapy is not included within immunosuppression regimens listed within EORTC-MSGERC criteria [1]. Three patients had chronic pulmonary conditions, 2 had decompensated liver cirrhosis, 1 had myasthenia gravis, and 1 had newly diagnosed HIV and presented with AIDS-defining illness (CD4 count, 0 cells/mm^3^). A patient with rheumatoid arthritis was receiving long-term low-dose prednisolone daily (5 mg).

Case 2 was admitted to the ICU but did not fulfill the diagnosis of putative invasive pulmonary aspergillosis (IPA) by Blot et al [4]. Case 10 received corticosteroid but at a dose lower than the required threshold to meet EORTC-MSGERC criteria. Case 14 had underlying myelodysplasia but was not neutropenic and was not receiving active therapy.

### Group b: Cases With an Isolated Single *Aspergillus* PCR

Twenty-two patients had established host factors and radiologic evidence suggestive of IA but with only a single positive *Aspergillus* PCR result from BALF samples, therefore lacking the mycologic criteria for defining IA [1] (Supplementary Table 2).

Sixteen patients in this group (73%) had an underlying hematologic malignancy (HM), of whom 6 (37%) were neutropenic (neutrophil count, ≤0.5 ×10^9^/L) and 3 (19%) underwent allogeneic stem cell transplantation. All demonstrated pulmonary nodules and/or consolidation on chest CT, with ground glass halos reported in 3. Seven (44%) had received mold-active prophylaxis.

Three (12%) were renal transplant recipients. Case 16 had PCR-proven influenza and received significant doses of corticosteroids, as outlined in EORTC-MSGERC [1]. However, EORTC-MSGERC mycologic criteria and the published case definition for influenza-associated pulmonary aspergillosis [6] were not met. One patient with systemic sclerosis and autologous stem cell transplantation was receiving corticosteroids, cyclophosphamide, and antithymocyte globulin (case 31).

### Group c: Cases With Nonspecific Findings on Chest CT

Seven patients had mycologic evidence of IA with a high serum GM (n = 3) or BALF GM (n = 4; Supplementary Table 3). The range of serum GM and BALF GM ODIs were 1.12 to 7.5 and 1.42 to 7.74, respectively. Six patients had an underlying HM, of which 4 were also allogeneic stem cell transplantation recipients; 1 patient had underlying chronic granulomatous disease.

All 7 patients either had nonspecific findings such as peribronchiolar inflammatory infiltrates, tree-in-bud, or ground glass changes on chest CT or positron emission tomography (PET) scan (n = 4) or did not undergo chest CT but had a chest radiograph only (n = 3). These findings do not fulfill current EORTC-MSGERC–defined features for IPA.

### **Group d: Cases With GM ODI** ≥**0.5 to <1.0 in BALF**

Three patients had an underlying HM and radiologic findings suggestive of IPA but had GM ODI from BALF ≥0.5 to <1.0 (Supplementary Table 4): specifically, ODIs of 0.6 (case 45), 0.9 (case 46), and 0.8 (case 47). For these cases, *Aspergillus* was not cultured from BALF. Case 47 had *Aspergillus* PCR detected in BALF.

### Antifungal Treatment

Treatment was initiated in 42 patients (89%): 41 (98%) received antifungal therapy alone, and 1 had antifungal therapy and functional endoscopic sinus surgery. The overall median duration of antifungal therapy was 99 days (IQR, 46–171). When each group was evaluated individually, 11 patients (73%) from group a, 22 (100%) from group b, 6 from group c (86%), and 3 (100%) from group d received treatment.

Thirty-nine patients (93%) received at least 7 days of a single primary antifungal agent; 2 (5%) died after receiving 3 and 5 days of antifungal therapy; and the remaining 1 patient received combination therapy (liposomal amphotericin B with posaconazole). Of those receiving a single agent, voriconazole monotherapy was the most prescribed primary antifungal agent (n = 28, 72%) for a median 88 days (IQR, 25–168).

### Mortality Outcome

All-cause 90-day mortality for the 47 patients was 33%. The median time from diagnosis of IA to death was 22 days (IQR, 10–46). All-cause 30- and 180-day mortality was 17% and 45%, respectively. The all-cause 90-day mortality of the study cohort with proven/probable IA (n = 221) was 30%, and the 30- and 180-day mortality was 18% and 35% (Table 1) [2].

**Table 1.** Mortality Outcomes and Intensive Care Unit Admissions Between Current Cases (n = 47) and Original Cohort of Proven/Probable Invasive Aspergillosis (n = 221) [2]

	Current Cases Not Meeting International Consensus Definitions (n = 47)	Previous Proven/Probable Cases of Invasive Aspergillosis (n = 221)
Primary outcome		
All-cause 90-d mortality	14 (33)^a^	67 (30)
Secondary outcomes		
All-cause 30-d mortality	8 (17)	40 (18)
All-cause 180-d mortality	18 (45)^b^	78 (35)
Intensive care unit admission	7 (15)	47 (21)
Patients with hematologic malignancy only		
All-cause 90-d mortality	10/26 (43)^c^	42/110 (38)

Data are presented as No. (%).

^a^Five patients were lost to follow-up prior to 90 days; hence, the denominator was 42.

^b^Seven patients were lost to follow-up prior to 180 days; hence, the denominator was 40.

^c^Three patients were lost to follow-up prior to 90 days; hence, the denominator was 23.

For patients with an underlying HM in the current study (n = 26), the 90-day mortality was 43%, whereas the 90-day mortality of the cohort of 110 patients with an HM with proven/probable IA was 38% (Table 1) [2].

## DISCUSSION

We provide insights into a cohort of 47 significant IA cases in Australia and New Zealand, which did not fulfill current internationally accepted case definitions for IA. The most recent iteration of EORTC-MSGERC in the diagnosis of invasive fungal disease was published in 2019, which takes into consideration *Aspergillus* PCR as a mycologic criterion and prespecified threshold for GM antigen in serum and/or BALF [1, 7]. We followed these criteria and recommendations in case classification and observed the exclusion of 47 of 382 cases that were significant, highlighting some of the difficulties in using consensus definitions for IA in the real-world setting. As such cases are often excluded from clinical trials, patients may not be offered new effective therapies and may experience worse outcomes. Notably, antifungal therapy was initiated in 89% of these patients, for a median 99 days. All-cause 90-day mortality was similar to patients with proven/probable IA (n = 221), according to recently published data (33% vs 30%), as were 30- and 180-day mortality (17% vs 18% and 45% vs 35%, respectively) and 90-day mortality for patients with an HM (43% vs 38%) [2] (Table 1).

Acknowledging the limitations of the EORTC-MSGERC and other international consensus definitions, numerous published reports have employed either modified EORTC-MSGERC criteria or algorithms for use in patients who are critically ill [4] to highlight the significant risk and poor outcomes in patients without established host factors who acquired IA—namely, those with solid tumors [8–11], liver failure/cirrhosis [12–20], and HIV [21, 22]. In 2 recent studies [9, 11], the overall 12-week mortality in patients with solid organ tumors and IA was significant at 33.0% and 56.9%, respectively. This mortality rate was comparable to that of those with HM/stem cell transplantation and IA (33% vs 40% and 56.9% vs 40.9%). Likewise, patients with liver failure/cirrhosis have been recognized to be at increased risk of developing IA due to cirrhosis-associated immune dysfunction affecting innate and adaptive immunity [23], with a mortality rate exceeding 70% as compared with patients with liver failure/cirrhosis but without IA [12–16, 18]. The 2 patients in our cohort with decompensated liver disease died within 90 days of IA diagnosis. Low clinical suspicion of IA in patients with solid organ cancer and liver failure likely led to delayed diagnosis of infection and treatment, resulting in poor outcomes [9, 13].

With the introduction of combination antiretroviral therapy, aspergillosis is now rare in HIV/AIDS [24]. It is, however, still important for clinicians to be aware of this potentially fatal infection in these patients. In a review of 859 patients by Denning and Morgan [21], the majority had CD4 counts <50 cells/mm^3^. A French series of 242 cases of IA over 20 years demonstrated that although overall 3-month mortality postdiagnosis of IA has improved over time, from approximately 70% in the era before voriconazole and combination antiretroviral therapy to 30% more recently, there was no mortality difference between patients with HIV/AIDS who had proven/probable IA based on EORTC-MSGERC and those who did not fulfill the criteria (49% of cohort; hazard ratio, 1.2; 95% CI, 0.7–1.8) [22], highlighting the clinical significance of cases not meeting criteria for EORTC-MSGERC definitions.

In recognition of these host factors that do not fall within the definitions, for patients with compatible clinical features of invasive fungal disease in the ICU, decompensated liver cirrhosis, solid tumors, moderate/severe chronic obstructive pulmonary disease, uncontrolled HIV infection with a CD4 cell count <200/mm^3^, and recent influenza or COVID-19 diagnosis were included as host factors in the recent Invasive Fungal Diseases in Adult Patients in Intensive Care Unit (FUNDICU) consensus [25], similar to our patient profile in this study cohort. The FUNDICU consensus was not published during our adjudication process for case inclusion of proven/probable IA. Nonetheless, if we were to apply these criteria to our group of patients (IA cases without host factors), 2 of 3 patients admitted to the ICU without host factors would have fulfilled the FUNDICU consensus criteria: patient 7 with AIDS-defining illness and a CD4 cell count <200/mm^3^ and patient 8 with decompensated liver cirrhosis. Notably, even though this was intended for use in clinical research, clinicians should be cognizant of these nonclassical host factors, which place patients at increased risk of acquiring IPA outside the ICU.

Updated EORTC-MSGERC definitions now include positive *Aspergillus* PCR as one of the mycologic criteria [1]. During the adjudication process for our series, 22 cases with isolated *Aspergillus* PCR were not regarded as probable cases, as EORTC-MSGERC requires at least 2 consecutive positive PCRs from blood samples or ≥2 duplicate positive PCR results from BALF to increase the specificity of the diagnosis. We concur with others [26] who have highlighted the incomplete clarity of the requirement for “2 or more duplicate PCR tests positive from BALF”—specifically, whether this represents positive PCR results from 2 different bronchoscopies, sampling from 2 different lobes during a single procedure, or duplicate runs on a single sample. Given the invasive nature of bronchoscopy, Huygens et al interpreted this new criterion as “2 positive PCRs on a single BALF sample” [26]. The Australasian mycology reference laboratories that performed the *Aspergillus* PCR as part of our study performed each test in duplicate before verification of results. This *Aspergillus* PCR assay is an in-house, validated, real-time PCR assay that targets the D1-D2 variable region of the 28S rDNA gene and simultaneously detects human B globin, which serves as an endogenous internal control [27, 28]. It is genus specific; that is, it detects all *Aspergillus* species but cannot discriminate among species. However, it is worth noting that the most frequent *Aspergillus* species causing invasive infections in Australia were *Aspergillus* section Fumigati [2, 29]. Clarification regarding this mycologic requirement for 2 positive PCRs from BALF is needed.

We did not capture cycle threshold values of the positive PCR results, which may have provided insights regarding the burden of disease and differentiating colonization from invasive disease [26, 30, 31]. Nevertheless, all patients in this group underwent antifungal treatment for a median 99 days, likely because the majority (86%) had an underlying HM, allogeneic stem cell transplantation, or solid organ transplant, where untreated IA would have adverse outcomes. The mortality outcomes of this subgroup were also similar when compared with the primary cohort of 221 IA cases (data not shown).

[^18^F]Fluorodeoxyglucose (FDG) PET/CT has been shown to be beneficial in diagnosing and managing invasive fungal disease in patients with cancer [32, 33]. Our patient's PET/CT (case 39) had progressive and intense FDG uptake in the right lung. Despite not having typical radiographic features of nodules or consolidation on chest CT, this intense FDG uptake is suggestive of IPA, especially given a GM BALF ODI of 2.5. With the increasing use and availability of newer imaging modalities such as FDG PET/CT, further studies based on prespecified criteria are needed to explore their role in confirming IA.

Our study was limited by the retrospective nature of case review and relatively small patient numbers. Our findings, however, highlight the limitations of current consensus definitions of invasive fungal disease in real-world clinical practice, offering the opportunity to review and revisit the EORTC-MSGERC host and mycology criteria for case inclusion, especially cases with only a single positive *Aspergillus* PCR result but with compatible host and radiologic evidence of IA.

In conclusion, clinicians need to be aware of the development of IA in hosts who do not fulfill the EORTC-MSGERC criteria and to initiate prompt investigation and management, given similar mortality to those diagnosed with proven/probable IA. The recent FUNDICU project has included additional host factors for nonneutropenic cases without classical risk factors but only for the ICU setting. The inclusion of *Aspergillus* PCR to the EORTC-MSGERC is a beneficial addition, but clarification regarding clinical application of this criterion is required. Studies are warranted, especially on cases with an isolated positive *Aspergillus* PCR result and its position in the assignment for probability of IA.

## Supplementary Data

Supplementary materials are available at *Open Forum Infectious Diseases* online. Consisting of data provided by the authors to benefit the reader, the posted materials are not copyedited and are the sole responsibility of the authors, so questions or comments should be addressed to the corresponding author.

## Supplementary Material

ofae594_Supplementary_Data

## Notes

***Acknowledgments*.** We would like to thank all members of the Australia New Zealand Mycoses Interest Group, Australia, for endorsing the study. We also thank Kate Hamilton, previously from the Centre for Infectious Diseases and Microbiology Laboratory Services, Institute of Clinical Pathology and Medical Research, New South Wales Health, who was a research coordinator and assisted with data collection and organization of adjudication meetings. We also thank Michelle England and Dianne J. Gardam from Fiona Stanley Hospital, Western Australia, who assisted with data collection, case ascertainment, and mycologic identifications, and Dr Bernard Hudson and Kerry Weeks from Royal North Shore Hospital, New South Wales, who also assisted with identification of cases and mycologic identifications.

***Patient consent statement***. The study was conducted under the auspices of the Australian and New Zealand Mycoses Interest Group. All study centers had Human Research Ethics Committee approval (coordinating HREC 2019/ETH11822) and waived the need of individual patient consent.

***Financial support.*** This work is not funded.
