# Supplementary material for: Identifying Gaps in the International Consensus Case Definitions for Invasive Aspergillosis: A Review of Clinical Cases Not Meeting These Definitions
Source: Open Forum Infect Dis. 2024 Oct 9;11(11):ofae594. doi: 10.1093/ofid/ofae594 (PMC11530957; doi:10.1093/ofid/ofae594)
Supplement: ofae594_Supplementary_Data [file ofae594_supplementary_data.docx]

**Supplementary Document**

Table S1. Cases lacking in host factors as specified on accepted criteria.

| **Case**  **No.** | **Age, gender** | **Underlying disease** | **Risks** | **Microbiology** | | | | **Imaging** | **Outcome at**  **90-day** | **International consensus not met** |
| --- | --- | --- | --- | --- | --- | --- | --- | --- | --- | --- |
|  |  |  |  | **Culture (site)** | ***Aspergillus* PCR (site)** | ***Aspergillus* GM (Site)** | **Cytology/ Micro-scopy (site)** | **Main features e.g., nodules consolidation, infiltrate** |  |  |
| 1 | 51, M | Lung cancer not on therapy, asthma | Nil | Positive (BAL), *A. fumigatus* complex | Yes (BAL, once) | Yes (BAL ODI: 2.9) | Negative | 5mm left upper lobe nodule, increased to 14x14mm cavitation within thin wall and localised lateral consolidation query fungal infection | Alive | EORTC/ MSGERC host factors (1) |
| 2 | 62, M | Lung disease: No formal diagnosis but CT chest showed evidence of previous tuberculosis exposure in lung; nil other diseases | ICU admission | Positive (BAL), *A. niger* complex | Yes (BAL, once) | Yes (BAL ODI: 6.3) | No positive cytological smear showing branching hyphae | Lung: Nodules, consolidation, multiple cavities, pleural thickening, fibrosis | Died | EORTC/ MSGERC host factors  Blot et al criteria on IPA in critically ill patients (required positive cytological smear showing branching hyphae, no host factors as well) (2) |
| 3 | 60, F | Thyroid cancer | Lenvatinib (kinase inhibitor) | Positive (BAL), *A. fumigatus* complex | Yes (BAL, once) | Negative  (serum ODI: 0.1) | Negative | Lung: Interval development of patchy & dense consolidation; single cavity | Alive | EORTC/ MSGERC host factors |
| 4 | 68, M | Non-small cell lung cancer, smoker | Construc-tion work in/near hospital | Negative | Negative | Yes (BAL ODI: 1.72) | Negative | Lung: An area of cavitation at left upper lobe has enlarged, with mediastinal extension. New scattered areas of centrilobular opacity in left lower lobe, likely infective/inflammatory in nature. | Alive | EORTC/ MSGERC host factors |
| 5 | 60, F | Asthma, smoker | Nil | Positive (BAL), *A. fumigatus* complex | Yes (BAL, once) | Yes (BAL ODI: 2.58) | Positive cytological smear showing branching hyphae | Lung: bilateral lung nodules, consolidation, single cavity | Alive | EORTC/ MSGERC host factors (1) |
| 6 | 61, F | Decompen-sated chronic liver disease | Nil | Negative | Yes (BAL, once) | Yes (BAL ODI: 1.8) | Negative | Lung: patchy ground glass changes with consolidation, multiple bilateral lung nodules | Died | EORTC/ MSGERC host factors |
| 7 | 60, M | HIV/AIDS (CD4 count=0), chronic liver disease, smoker | ICU admission | Positive (BAL), *A. fumigatus* complex | Yes (BAL, once) | Yes (BAL ODI: 5.3) | Positive micro-scopy from clinical specimen | Lung: tree-in-bud with ground glass nodularities; 3.6cm cavity in apical segment | Alive | EORTC/ MSGERC host factors  Blot et al criteria on IPA in critically ill patients (no host factors) (2) |
| 8 | 52, M | Chronic liver disease (decompen-sated) | ICU admission | Positive (BAL), *A. fumigatus* complex | Negative | Negative | Negative | Lung: two cavitating nodules in keeping with abscesses, consolidation | Died | EORTC/ MSGERC host factors  Blot et al criteria on IPA in critically ill patients (no host factors) (2) |
| 9 | 79, F | Bronchiecta-sis | Nil | Negative | Yes (BAL, once) | Yes (BAL ODI: 2.6) | Negative | Lung: Multifocal nodular areas of consolidation; largest nodule has surrounding halo | Alive | EORTC/ MSGERC host factors |
| 10 | 72, M | Non-small cell lung cancer (metastatic), granulomatosis with polyangiitis on prednisolone, diabetes, COPD | Cortico-steroids (0.27mg/kg/ day for <28 days) [dose needed to be ≥0.3 mg/kg for ≥3 weeks to fulfill EORTC/ MSGERC criteria] | Negative | Negative | Yes (BAL ODI: 3.67) | Negative | Lung: New nodules, new nodules, one with cavitation | Alive | EORTC/ MSGERC host factors  Bulpa et al criteria on IPA in patients with chronic obstructive pulmonary disease (patient needed to have positive *Aspergillus* culture and/or microscopy from BAL, OR positive serum antibody test for *A. fumigatus*, OR two consecutive positive serum GM tests) (3) |
| 11 | 37, F | Metastatic breast cancer not on therapy | Nil | Negative | Negative | Yes (BAL ODI: 6.7) | Negative | Lung: increased bilateral solid & ground glass nodularities | Alive | EORTC/ MSGERC host factors |
| 12 | 46, F | Small cell lung cancer not on chemotherapy | Nil | Positive (BAL), *A. fumigatus* complex | Negative | Negative | Negative | Lung: confluent consolidation with tree-in-bud nodules | Alive | EORTC/ MSGERC host factors |
| 13 | 60, M | Myasthenia gravis | Nil | Positive (BAL), *A. fumigatus* complex | Negative | Yes (BAL ODI: unknown) | Negative | HRCT: large nodular infiltrates | Died | EORTC/ MSGERC host factors |
| 14 | 80, M | Myelodysplasia (not neutropenic, not on therapy) | Nil | Positive (induced sputum), *A. fumigatus* complex | Negative | Negative | Negative | Lung: Mass-like consolidation, bilateral pulmonary nodules with surrounding ground glass opacities | Died | EORTC/ MSGERC host factors |
| 15 | 67, M | Rheumatoid arthritis | Low dose predniso-lone 5mg daily | Negative | Negative | Yes (serum ODI: 0.95) | Negative | Lung: large thick-walled cavitatory lesion, additional solid and ground glass nodules scattered throughout both lungs | Alive | EORTC/ MSGERC host factors |

GM = galactomannan; BAL = bronchoalveolar lavage; ODI = optical density index; ICU = intensive care unit; HIV = human immunodeficiency virus; AIDS = acquired immunodeficiency syndrome; COPD = chronic obstructive pulmonary disease; EORTC/MSGERC = European Organization

for Research and Treatment of Cancer and the Mycoses Study Group Education and Research Consortium; IPA = invasive pulmonary aspergillosis; HRCT = high resolution computed tomography

Table S2. Cases with only isolated/single positive *Aspergillus* PCR.

| **Case no.** | **Age, gender** | **Underlying disease** | **Risks** | **Microbiology** | | | | **Imaging** | **Outcome at 90-day** | **International consensus not met** |
| --- | --- | --- | --- | --- | --- | --- | --- | --- | --- | --- |
|  |  |  |  | **Culture (site)** | ***Aspergillus* PCR (site)** | ***Aspergillus* GM (Site)** | **Cytology/ Micro-scopy (site)** | **Main features e.g. nodules consolidation, infiltrate** |  |  |
| 16 | 64, M | ABPA, Bronchiecta-sis, PCR positive influenza <3 days | Corticoste-roids  >0.3mg/kg/day for >3 months | Negative | Yes (BAL, once) | Negative | Positive microscopy from clinical specimen | Lung: New tree-in-bud infiltrates and patchy consolidation in addition to known existing aspergilloma & bronchiectasis (new changes are in different parts of the lung fields from aspergilloma and bronchiectasis) | Alive | EORTC/ MSGERC mycological evidence (1)  Verweij et al criteria on influenza-associated pulmonary aspergillosis (IAPA) in ICU patients (patient needed to have positive BAL culture, OR serum GM >0.5, OR BAL GM $\geq$1.0, there’s no mention of *Aspergillus* PCR) (4) |
| 17 | 68, M | Myelodyspla-sia, allogeneic HSCT, asthma | Chemotherapy, | Negative | Yes (BAL, once) | Negative | Negative | Lung: multifocal nodules most likely representing fungal infection | Died | EORTC/ MSGERC mycological evidence (1) |
| 18 | 69, M | Myelodyspla-sia transformed to AML, allogeneic HSCT | Nil | Negative | Yes (BAL, once) | Negative | Negative | Lung: A solid stellate soft tissue density nodule is seen in the left lower lobe; reticular nodular interstitial densities and thickening is also seen | Alive  (but died at day 111) | EORTC/ MSGERC mycological evidence |
| 19 | 41, M | Myelodys-plastic syndrome | Growth factors, neutropenia | Negative | Yes (BAL, once) | Negative  (BAL ODI: 0.2) | Negative | Lung: multiple new pulmonary nodules, lobar infiltrate | Alive  (but died at day 132) | EORTC/ MSGERC mycological evidence |
| 20 | 78, M | Leukaemia, ex-smoker | Zanubruti-nib (BTK inhibitor) | Negative | Yes (BAL, once) | Negative | Negative | Lung: Increase in size of the subsolid left upper lobe lung nodule | Alive | EORTC/ MSGERC mycological evidence |
| 21 | 49, M | Primary central nervous system lymphoma | Chemotherapy, neutropenia | Negative | Yes (BAL, once) | Negative | Negative | Lung: numerous scattered nodular opacities; another larger mass with a similar halo of ground-glass changes around it | Alive | EORTC/ MSGERC mycological evidence |
| 22 | 75, F | Relapsed/ refractory leukaemia | Chemothe-rapy, neutropenia | Negative | Yes (BAL, once) | Negative | Negative | Lung: Peripheral right middle lobe: ground-glass opacity most likely representing infection and with fungal infection possible. | Died | EORTC/ MSGERC mycological evidence |
| 23 | 73, M | Non-small cell lung cancer | Cortico-steroids; Chemothe-rapy; Checkpoint inhibitor -pembroli-zumab | Negative | Yes (BAL, once) | Yes, according to manufacturer’s cut off (BAL ODI: 0.52) | Negative | Lung: Nodules; New ground glass changes | Alive | EORTC/ MSGERC mycological evidence |
| 24 | 61, M | Relapsed/ refractory lymphoma | Cortico-steroids, cytarabine, methotre-xate | Negative | Yes (BAL, once) | Negative  (BAL ODI: 0.38) | Negative | Lung: new nodular pulmonary lesions | Alive | EORTC/ MSGERC mycological evidence |
| 25 | 64, F | Lymphoma, COPD | Rituximab, chemothe-rapy | Negative | Yes (BAL, once) | Negative  (BAL ODI: 0.07) | Negative | Lung: Focus of intensely avid RUL consolidation on PET/CT | Alive | EORTC/ MSGERC mycological evidence |
| 26 | 50, M | Myelodyspla-sia | Growth factors, neutropenia, ICU admission | Negative | Yes (BAL, once) | Negative | Negative | Lung: Consolidation with areas of central necrosis within the right lower lobes | Died | EORTC/ MSGERC mycological evidence  Blot et al criteria on IPA in critically ill patients (no mention of *Aspergillus* PCR in this algorithm) (2) |
| 27 | 64, M | Leukaemia | Cortico-steroids, chemothe-rapy | Negative | Yes (BAL, once) | Yes (BAL ODI: unknown) | Negative | Lung: Multifocal areas of consolidation in both lungs | Lost to follow up | EORTC/ MSGERC mycological evidence |
| 28 | 76, M | Lymphoma, diabetes | Chemothe-rapy | Negative | Yes (BAL, once) | Negative  (BAL ODI: 0.01) | Negative | Lung: New multifocal consolidation, tree-in-bud nodules, consolidation with a ground-glass halo | Died | EORTC/ MSGERC mycological evidence |
| 29 | 65, M | Transformed AML from MDS | Chemotherapy, neutropenia | Negative | Yes (BAL, once) | Negative  (BAL ODI: 0.01) | Negative | Lung: nodules, consolidation, lobar infiltrate | Lost to follow up | EORTC/ MSGERC mycological evidence |
| 30 | 52, M | Kidney transplant | Cortico-steroids, Mycophe-nolate, Tacrolimus | Negative | Yes (BAL, once) | Negative | Negative | Lung: Progressive frank consolidation | Alive | EORTC/ MSGERC mycological evidence |
| 31 | 52, F | Systemic sclerosis, autologous HSCT | Cortico-steroids, Cyclophos-phamide, ATG, ICU admission | Negative | Yes (BAL, once) | Negative | Negative | Lung: Cavitating lesion with surrounding consolidation | Alive (but died at day 148) | EORTC/ MSGERC mycological evidence  Blot et al criteria on IPA in critically ill patients (no mention of *Aspergillus* PCR in this algorithm) (2) |
| 32 | 70, M | Myelodysplasia | Chemothe-rapy, neutropenia | Negative | Yes (BAL, once) | Negative | Negative | Lung: Multiple solid nodules and ground-glass nodular changes; dense consolidation | Died | EORTC/ MSGERC mycological evidence |
| 33 | 68, M | Kidney transplant, dialysis, diabetes | Cortico-steroids, Mycophe-nolate, Tacrolimus | Negative | Yes (BAL, once) | Negative | Negative | Lung: Lobulated mass like opacities with air-bronchograms and small nodules in the right lung | Alive | EORTC/ MSGERC mycological evidence |
| 34 | 29, M | Allogeneic HSCT | Chemothe-rapy | Negative | Yes (BAL, once) | Negative | Negative | Lung: Multifocal peri-bronchovascular consolidation with surrounding ground glass opacities | Lost to follow up | EORTC/ MSGERC mycological evidence |
| 35 | 60, M | Leukaemia | Monoclonal antibody; chemothe-rapy, checkpoint inhibitor | Negative | Yes (BAL, once) | Negative | Negative | Lung: consolidation; peri-bronchial ground glass opacifications right lower lobe | Alive | EORTC/ MSGERC mycological evidence |
| 36 | 61, M | Kidney transplant, diabetes | Cortico-steroids, tacrolimus, mycophe-nolate | Negative | Yes (BAL, once) | Negative | Negative | Lung: nodules, single cavity. Cavitating lesion right upper lobe. Tree-in-bud ground-glass nodules and ground glass opacity within the right middle lobe | Alive | EORTC/ MSGERC mycological evidence |
| 37 | 70, M | Leukaemia (relapsed/ refractory) | Chemothe-rapy; Bcl-2 Inhibitor | Negative | Yes (BAL, once) | Negative | Negative | Lung: New focal opacities at upper lobes with surrounding ground glass halo | Alive (but died at day 144) | EORTC/ MSGERC mycological evidence |

GM = galactomannan; BAL = bronchoalveolar lavage; ABPA = allergic bronchopulmonary aspergillosis; HSCT = haematopoietic stem cell transplant; AML = acute myeloid leukaemia; GVHD = graft versus host disease; BTK = bruton tyrosine kinase; COPD = chronic obstructive pulmonary disease; ODI = optical density index; PET/CT = positron emission tomography/computer tomography; MDS = myelodysplastic syndrome; ATG = anti-thymocyte globulin; EORTC/MSGERC = European Organization for Research and Treatment of Cancer and the Mycoses Study Group Education and Research Consortium

Table S3. Cases lacking pre-specified patterns on chest computed tomography (CT) based on accepted definitions.

| **Case no.** | **Age, gender** | **Underlying disease** | **Risks** | **Microbiology** | | | | **Imaging** | **Outcome at 90-day** | **International consensus not met** |
| --- | --- | --- | --- | --- | --- | --- | --- | --- | --- | --- |
|  |  |  |  | **Culture (site)** | ***Aspergillus* PCR (site)** | ***Aspergillus* GM (Site)** | **Cytology/ Microscopy (site)** | **Main features e.g. nodules consolidation, infiltrate** |  |  |
| 38 | 36, F | Relapsed leukaemia, allogeneic HSCT | ICU admission, corticosteroids, mycophenolate, chemotherapy | Negative | Yes (BAL) | Yes (serum ODI: 7.5) | Negative | Lung: consolidation (chest X-ray only). No CT chest performed as patient was too unwell and died shortly after diagnosis of relapsed leukaemia | Died | EORTC/ MSGERC clinical features (1) |
| 39 | 46, F | Refractory leukaemia, rheumatoid arthritis (not on active treatment) | Chemotherapy, neutropenia | Negative | Negative | Yes (BAL ODI: 2.5) | Negative | PET/CT: Intense fludeoxyglucose F18 (FDG) uptake at right inferior hilum and along segmental bronchial thickening into adjacent right lower lobe. This uptake has significantly progressed since previous PET/CT, and may represent an infection  CT chest: The perihilar thickening and changes in the right lower hilum, adjacent to the surgical clips remain roughly unchanged on this non-contrast CT scan,  compared to the PET/CT | Alive | EORTC/ MSGERC clinical features |
| 40 | 57, M | Relapsed/ refractory lymphoma, allogeneic HSCT | ICU admission, corticosteroids, mycophenolate mofetil, checkpoint inhibitor (Nivolumab) | Negative | Negative | Yes (BAL ODI: 3.51) | Negative | CT chest: Patchy ground glass, tree-in-bud opacities | Alive | EORTC/ MSGERC clinical features |
| 41 | 62, M | Allogeneic HSCT, diabetes | Corticosteroids, Mycophenolate | Positive (BAL), *A. fumigatus* complex, *A. niger* complex, *A. terreus* complex | Negative | Yes (BAL ODI: 7.74) | Negative | CT chest: Bilateral subpleural ground glass opacities, consistent with organising pneumonia | Alive | EORTC/ MSGERC clinical features |
| 42 | 34, M | Lymphoma | Neutropenia <30 days | Negative | Negative | Yes (serum ODI: 4.79) | Negative | Chest X-Ray: bilateral air space opacities; no CT chest done | Died | EORTC/ MSGERC clinical features |
| 43 | 30, M | Chronic granulomatosis disease | Nil | Negative | Negative | Yes (BAL ODI: 1.42) | Negative | Chest X-Ray only: patchy widespread opacification | Lost to follow up | EORTC/ MSGERC clinical features |
| 44 | 62, F | Lymphoma; allogeneic HSCT | corticosteroids, Filgrastim, chemotherapy (allogeneic HSCT conditioning) | Negative | Negative | Yes (serum ODI: 1.12) | Negative | CT chest: overall suggestive of cardiac failure, but there is a small focal area of peribronchiolar inflammatory infiltrate in the left upper lobe which may reflect superimposed infection | Died | EORTC/ MSGERC clinical features |

GM = galactomannan; BAL = bronchoalveolar lavage; ODI = optical density index; HSCT = haematopoietic stem cell transplant; ICU = intensive care unit; PET/CT = positron emission tomography/computed tomography; EORTC/MSGERC = European Organization for Research and Treatment of Cancer and the Mycoses Study Group Education and Research Consortium

Table S4. Cases with borderline galactomannan (GM) optical density index (ODI) between $\geq$0.5 to <1.0 in BAL.

| **Case no.** | **Age, gender** | **Underlying disease** | **Risks** | **Microbiology** | | | | **Imaging** | **Outcome at 90-day** | **International consensus definition not met** |
| --- | --- | --- | --- | --- | --- | --- | --- | --- | --- | --- |
|  |  |  |  | **Culture (site)** | ***Aspergillus* PCR (site)** | ***Aspergillus* GM (Site)** | **Cytology/ Microscopy (site)** | **Main features e.g. nodules consolidation, infiltrate** |  |  |
| 45 | 83, M | Myelodysplasia, diabetes | Neutropenia (30 days, nadir 0.3) | Negative | Negative | Yes (BAL GM ODI: 0.6) | Negative | Lung: Numerous nodular densities with surrounding ground glass changes | Died | EORTC/ MSGERC mycological evidence (1) |
| 46 | 58, M | Leukaemia, allogeneic HSCT | Cyclosporin | Negative | Negative | Yes (BAL GM ODI: 0.9) | Negative | Lung: New development of ground glass densities and small discrete nodules | Alive | EORTC/ MSGERC mycological evidence |
| 47 | 61, F | Acute promyelocytic leukaemia | Corticosteroids, Chemotherapy, neutropenia | Negative | Yes (BAL, once) | Yes (BAL GM ODI: 0.8) | Negative | Lung: Scattered diffuse nodules up to 12mm (which increased in size & numbers on serial scans), consolidation, lobar infiltrate | Lost to follow up | EORTC/ MSGERC mycological evidence |

GM = galactomannan; BAL = bronchoalveolar lavage; ODI = optical density index; HSCT = haematopoietic stem cell transplant; EORTC/MSGERC = European Organization for Research and Treatment of Cancer and the Mycoses Study Group Education and Research Consortium

**References:**

1. Donnelly JP, Chen SC, Kauffman CA, Steinbach WJ, Baddley JW, Verweij PE, et al. Revision and Update of the Consensus Definitions of Invasive Fungal Disease From the European Organization for Research and Treatment of Cancer and the Mycoses Study Group Education and Research Consortium. Clin Infect Dis. 2020;71(6):1367-76.

2. Blot SI, Taccone FS, Van den Abeele AM, Bulpa P, Meersseman W, Brusselaers N, et al. A clinical algorithm to diagnose invasive pulmonary aspergillosis in critically ill patients. Am J Respir Crit Care Med. 2012;186(1):56-64.

3. Bulpa P, Dive A, Sibille Y. Invasive pulmonary aspergillosis in patients with chronic obstructive pulmonary disease. Eur Respir J. 2007;30(4):782-800.

4. Verweij PE, Rijnders BJA, Bruggemann RJM, Azoulay E, Bassetti M, Blot S, et al. Review of influenza-associated pulmonary aspergillosis in ICU patients and proposal for a case definition: an expert opinion. Intensive Care Med. 2020;46(8):1524-35.
